# Screening of Alzheimer’s disease by facial complexion using artificial intelligence

**DOI:** 10.18632/aging.202545

**Published:** 2021-01-25

**Authors:** Yumi Umeda-Kameyama, Masashi Kameyama, Tomoki Tanaka, Bo-Kyung Son, Taro Kojima, Makoto Fukasawa, Tomomichi Iizuka, Sumito Ogawa, Katsuya Iijima, Masahiro Akishita

**Affiliations:** 1 Department of Geriatric Medicine, Graduate School of Medicine, The University of Tokyo, Tokyo, Japan; 2 Department of Diagnostic Radiology, Tokyo Metropolitan Geriatric Hospital and Institute of Gerontology, Tokyo, Japan; 3 Institute of Gerontology, The University of Tokyo, Tokyo, Japan; 4 Department of Nuclear Medicine, Fukujuji Hospital, Japan Anti-Tuberculosis Association, Kiyose, Japan; 5 Center for Dementia, Fukujuji Hospital, Japan Anti-Tuberculosis Association, Kiyose, Japan; 6 Institute for Future Initiatives, The University of Tokyo, Tokyo, Japan

**Keywords:** artificial intelligence, face, dementia, machine learning

## Abstract

Despite the increasing incidence and high morbidity associated with dementia, a simple, non-invasive, and inexpensive method of screening for dementia is yet to be discovered. This study aimed to examine whether artificial intelligence (AI) could distinguish between the faces of people with cognitive impairment and those without dementia.121 patients with cognitive impairment and 117 cognitively sound participants were recruited for the study. 5 deep learning models with 2 optimizers were tested. The binary differentiation of dementia / non-dementia facial image was expressed as a “Face AI score”. Xception with Adam was the model that showed the best performance. Overall sensitivity, specificity, and accuracy by the Xception AI system and AUC of the ROC curve were 87.31%, 94.57%, 92.56%, and 0.9717, respectively. Close and significant correlations were found between Face AI score and MMSE (*r* = −0.599, *p* < 0.0001). Significant correlation between Face AI score and chronological age was also found (*r* = 0.321, *p* < 0.0001). However, MMSE score showed significantly stronger correlation with Face AI score than chronological age (*p* < 0.0001). The study showed that deep learning programs such as Xception have the ability to differentiate the faces of patients with mild dementia from that of patients without dementia, paving the way for future studies into the development of a facial biomarker for dementia.

## INTRODUCTION

Dementia is one of the most serious problems facing a global aging population. Diagnosis of dementia is important for early intervention of the disease. However, many diagnostic methods are invasive or are time-consuming. For example, psychological assessment takes time, examination of cerebrospinal fluid is invasive and amyloid positron emission tomography is costly. Thus, there is demand for a simple, noninvasive, and inexpensive method for screening for dementia.

The perceived age of older adults was shown to be a robust biomarker of aging that is predictive of survival, telomere length [1], DNA methylation [2], carotid atherosclerosis [3], and bone status [4]. It was demonstrated that perceived age reflects cognitive function more closely than chronological age [5]. Furthermore, in the authors’ experience, advanced Alzheimer’s disease (AD) patients display a specific complexion. Thus, it was postulated that cognitive decline may be expressed in a patient’s face.

Deep learning, a major branch of machine learning in artificial intelligence (AI), has remarkably improved the performance and detection capabilities of AI programs since convolutional neural network (CNN) was first developed [6]. The authors have previously reported how CNN could be used to discern AD and dementia with Lewy bodies (DLB) perfusion single photon emission tomography (SPECT) images. The differentiation was made using cingulate island sign [7]. Based on this information, it was hypothesized that AI software may be able to classify patients as having cognitive impairment or not using facial recognition.

The present study aimed to examine whether AI can distinguish facial traits of cognitive impairment patients from that of non-dementia patients. The findings of this study lay the foundations for the development of a non-invasive, inexpensive and rapid screening tool for cognitive impairment using AI.

## RESULTS

### Demographics

3 patients from the Department of Geriatric Medicine were diagnosed as cognitively normal after several tests and were thus included in the non-dementia arm of the study. Although the participants from the Kashiwa cohort live self-sufficiently in the community, one participant showed declining MMSE over the past few years and was thus classified into the cognitive impairment arm of the study. Two patients were diagnosed to be dementia with Lewy bodies (DLB), two were diagnosed to idiopathic normal pressure hydrocephalus (iNPH), one had aphasia due to cerebral infarction. The rest of the participants were diagnosed to be Alzheimer’s disease. The five non-AD patients were excluded from the analyses.

Table 1 summarizes the demographics of participants.

**Table 1:** Demographic features of study participants.

	**Cognitive impairment**		**Normal**		**p**
	**Male**	**Female**	**Male**	**Female**	
Participants	121		117		
Sex	46	75	50	67	0.509 ^a^
Age	80.6±6.5	81.5±6.2	75.8±6.0	75.7±5.1	1.20 × 10^−9 b^
MMSE score	21.9±5.0	21.3±5.1	28.8±1.8	29.0±1.3	4.23 × 10^−34 b^
photographs	54	80	155	195	
hypertension (+/−)	26/19	57/19	31/19	38/29	0.139^a^
hyperlipidemia (+/−)	13/33	30/46	25/24	37/30	0.00603^a^
diabetes (+/−)	13/33	15/61	20/30	25/42	0.0114^a^
osteoporosis (+/−)	9/37	33/43	19/31	25/33	0.112^a^

Data are shown as numbers or means ± standard deviation. MMSE, mini-mental state examination; a, Fisher’s exact test comparing cognitive impairment and normal; b, one-way analysis of variance.

### Models examined

**Figure 1:**
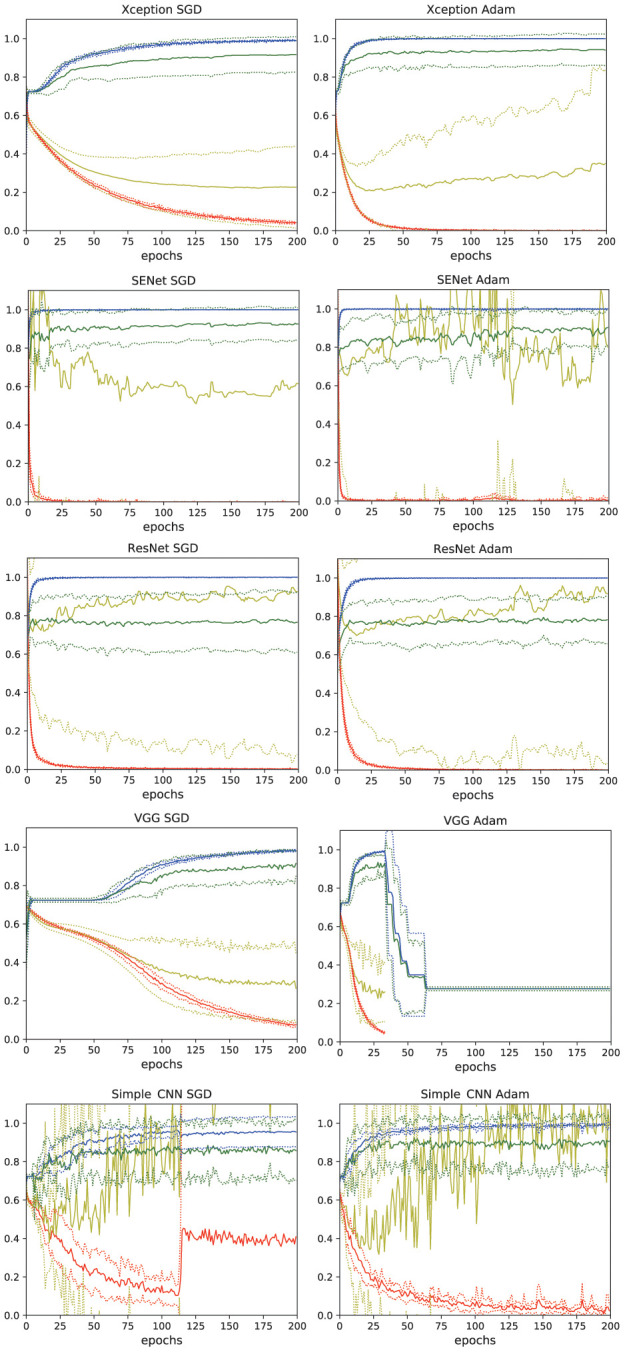
**Learning curves of the deep learning models.** Xception, SENet50, ResNet50, VGG16, and simple CNN with SGD and Adam optimizers were tested. The thin lines denote mean ± standard deviation of 10 groups. *blue*: accuracy, *red*: loss, *green*: validation accuracy, *yellow*: validation loss.

Learning curves for models, Xception, SENet50, ResNet50, VGG16, and simple CNN with SGD and Adam optimizer are shown in Figure 1. Xception with Adam showed the best performance with a peak validation accuracy of more than 94% and a bottom validation loss of less than 0.21. Xception with Adam optimizer showed slightly better performance, therefore Adam appeared to be the preferred optimizer for Xception. ResNet, SENet showed fast learning, however validation cross entropy loss did not drop adequately. VGG16 with Adam showed good accuracy, however cross entropy loss could not be calculated in an early epoch. VGG16 with SGD showed successful learning with the peak validation accuracy being approximately 91%. Simple CNN with Adam also showed learning with the peak validation accuracy being approximately 92%. However, validation cross entropy loss of simple CNN did not show stability.

From these results, Xception with Adam applied as an optimizer was chosen for use in this study. 28 epochs was chosen as this was where the bottom of loss was seen in the model.

### Evaluation of the AI model

Sensitivity, specificity, and accuracy by the AI system (boundary: Face AI score = 0) and area under the curve (AUC) of receiver operating characteristic (ROC) curve of the 10 groups were 87.52±11.91%, 94.57±10.88%, 92.56±8.22%, and 0.9827±0.0201, respectively (± denotes standard deviation). The best sensitivity and specificity according to ROC analysis were 97.80±3.36%, 96.00±3.43%, respectively.

**Figure 2:**
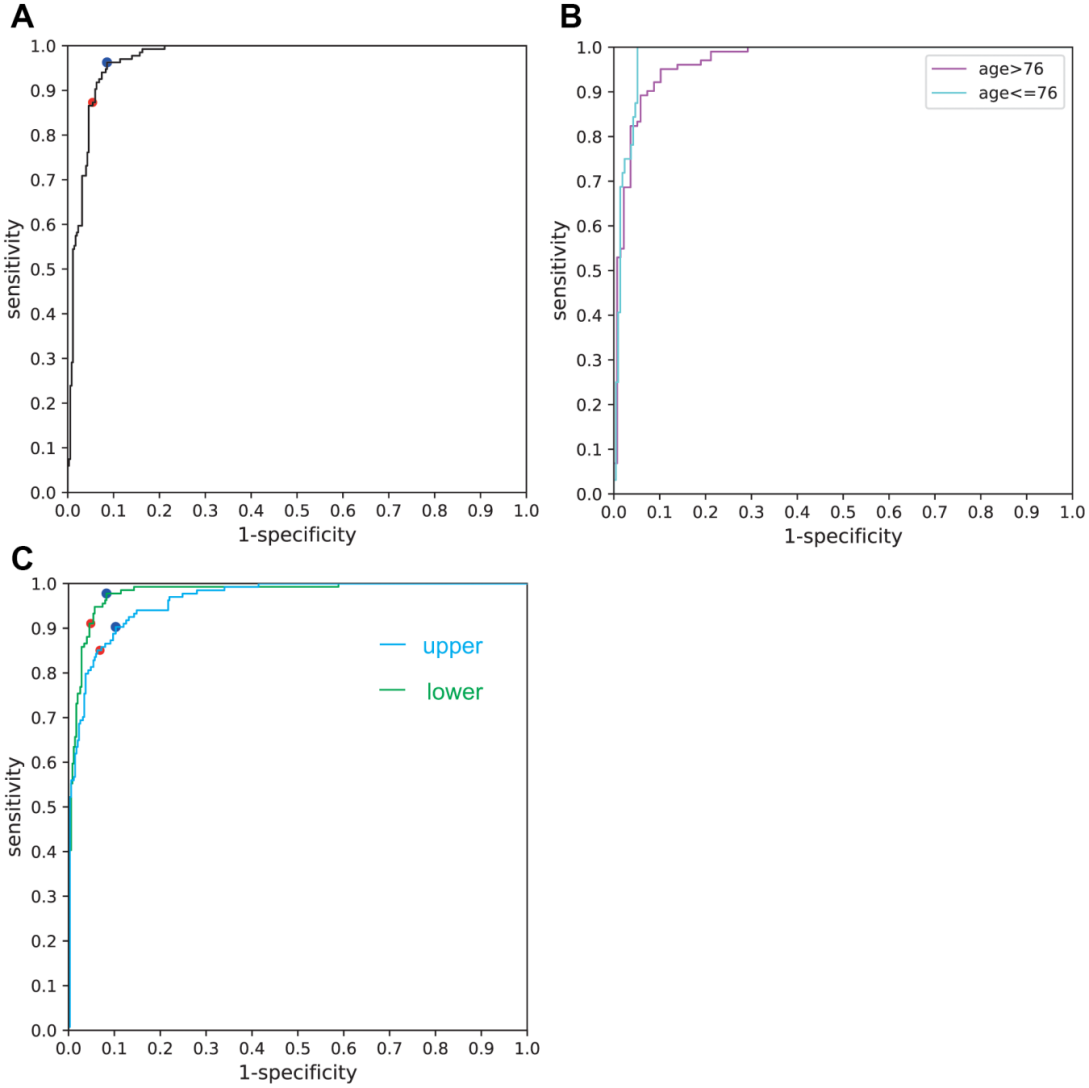
(**A**) ROC curve for the overall facial images used. The red dot denotes the sensitivity and specificity calculated by the AI software (boundary: Face AI score = 0). The blue dot denotes the sensitivity and specificity calculated by ROC analysis (boundary: Face AI score = −1.51). (**B**) ROC curve for the aged (>76) and the relatively young (≤76). (**C**) ROC curve for upper half and lower half faces.

Overall sensitivity, specificity, and accuracy of the Xception AI system (boundary: Face AI score = 0) and AUC of ROC curve were 87.31%, 94.57%,92.56%, and 0.9717, respectively (Table 2). The ROC curve is displayed in Figure 2A. Sensitivity and specificity according to ROC analysis were 96.27%, 91.42% (boundary: Face AI score = −1.51).

**Table 2:** Performance of AI.

	**Sensitivity**	**Specificity**	**Accuracy**	**AUC**	**Best sensitivity**	**Best specificity**	**Threshold**
total	0.8731	0.9457	0.9256	0.9717	0.9627	0.9143	-1.51
aged (>76)	0.8333	0.9489	0.8996	0.9687	0.9510	0.8978	-1.51
young (≤76)	1	0.9437	0.9510	0.9805	1	0.9484	0.04
upper face	0.8507	0.9314	0.9091	0.9635	0.9030	0.8971	-0.65
lower face	0.9104	0.9514	0.9401	0.9803	0.9776	0.9171	-1.2

Median age of the participants was 76. Sensitivity, specificity, accuracy and AUC of ROC curve for the aged (>76) were 83.33%, 94.89%, 89.96%, and 0.9687, respectively; those for the relatively young (≤76) were 100%, 94.37%, 95.10%, 0.9805, respectively (Figure 2B) (Table 2).

### Correlation between face AI score and MMSE/age

**Figure 3:**
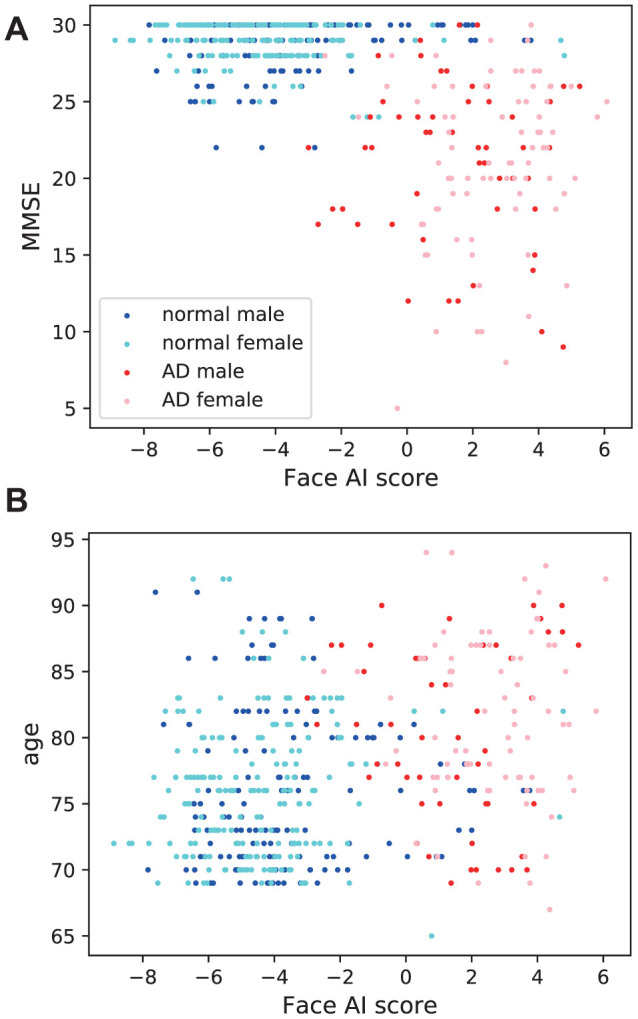
Association of Face AI score with (**A**) MMSE and (**B**) chronological age. Face AI score correlated closely with (a) MMSE (*r* = −0.599, *t* = −16.40, *p* = 2.47×10^−48^) and relatively weakly with (b) age (*r* = 0.321, *t* = 7.44, *p* = 4.57 × 10^−13^). The Steiger’s test found the difference in correlation coefficients to be significant (*p* = 3.25 × 10^−35^).

Significant close correlations were found between Face AI score and MMSE (*r* = −0.599, *t* = −16.40, *p* = 2.47 × 10^−48^; Figure 3A). Significant correlation between Face AI score and chronological age was also found (*r* = 0.321, *t* = 7.44, *p* = 4.57 × 10^−13^; Figure 3B). However, MMSE score showed a significantly stronger correlation with Face AI score than chronological age (*t* = 13.45, *p* = 3.25 × 10^−35^; Steiger’s test [8]).

The correlation coefficient between MMSE and Face AI score in female (*r* = −0.661) was significantly stronger than that in male (*r* = −0.501) (*p* = 0.00833) when evaluated using Fisher’s *Z*-transformation method [9].

The difference in the correlation coefficient between age and Face AI score by sex (male: *r* = 0.229, female: *r* = 0.388) when assessed using the same method was not significant (*p* = 0.0565).

### Analysis of upper and lower half faces

44 epochs was chosen for upper half and 41 epochs for lower half, as these were where the bottom of loss was seen in the model.

Sensitivity, specificity, accuracy and AUC of ROC curve for upper half faces were 85.07%, 93.14%, 90.91%, and 0.9635, respectively; those for the lower half faces were 91.04%, 95.14%, 94.01%, 0.9803, respectively (Figure 2C) (Table 2).

## DISCUSSION

The deep learning network implemented in this study successfully discerned faces of participants with cognitive impairment from those without dementia. With an accuracy of 92.56% and AUC of ROC being 0.9717, this method is reliable enough for implementation as an initial screening test for dementia. The relatively low sensitivity (87.31%), which may be attributable to the fewer number of dementia participant facial images, could be improved to 96.27% by employing ROC analysis. Face AI score correlated significantly more strongly with MMSE than with age. The weaker correlation between Face AI score and age (*r* = 0.321) would imply that the significant difference between age of dementia participants with and without dementia is unlikely to have affected the AI system. If the AI system relied on participants age, limiting the age range would worsen the results. However, limiting the age did not impair the accuracy (Figure 2B).

The AI system is too complicated and has so-called “black box” nature. Although both upper and lower half faces showed excellent performance, lower half showed slightly better performance than upper half contrary to our expectation (Figure 2C). The system may get more information from mouths or wrinkles than eyes or hair.

The correlation coefficient between Face AI score and MMSE was significantly higher in females than in males. A previous study by the authors demonstrated a significantly stronger correlation between MMSE and perceived age than with chronological age but only in female participants [5], which may be attributable to the tendency for cognitively healthy older women to pay more attention to their appearances relative to their cognitively impaired counterparts, resulting in a marked difference in perceived age.

The fact that SENet50, ResNet50, and simple CNN models were unable to reduce loss during discriminating facial images of dementia and non-dementia participants suggests that the task is more difficult for AI software than discerning differences in SPECT images [7]. This is understandable given that while SPECT images can be classified by trained nuclear medicine physicians, diagnosis of dementia from facial images alone cannot be done manually. Suitable deep learning networks such as Xception are required to accomplish the complex task of detection of dementia through facial images.

This study has several limitations. Firstly, the study comprised only 484 images as it was performed at a single institution with a single cohort. Moreover, this study may be affected by institutional biases. Although pretraining assisted AI system learning with limited images, further studies employing a larger number of images from multicenter will be needed to confirm the results. Secondly, the non-dementia participants of this study may have had undetected dementia that did not require nursing care. Similarly, though the majority of dementia participants were assumed to have Alzheimer's disease, this was not confirmed using pathology or amyloid positron emission tomography, and one participant from Kashiwa cohort did not underwent sufficient tests. Thus, some participants may have been suffering other forms of dementia such as DLB, vascular dementia or normal pressure hydrocephalus. The facial differentiation of DLB and AD patients may be an interesting study in the future. Finally, the facial images included in this study were only front facing images of Japanese participants with neutral expressions. The ability to use images from various angles, of people from varied ethnicities and with a variety of facial expressions would improve the robustness of the AI system.

## CONCLUSIONS

The study showed that deep learning software such as Xception has the ability to differentiate facial images of people with mild dementia from those of people without dementia. This may pave the way for the clinical use of facial images as a biomarker of dementia.

## MATERIALS AND METHODS

### Participants

Dementia patients were recruited mainly from the Department of Geriatric Medicine, The University of Tokyo Hospital. Many of them also participated in a previous perceived age study [5]. The majority of participants without dementia were recruited from the Kashiwa cohort organized by the Institute of Gerontology, The University of Tokyo. Patients from the Department of Geriatric Medicine who were diagnosed as cognitively normal after several tests were included in the non-dementia arm of the study. Participants from the Kashiwa cohort who showed declining MMSE over the past few years were classified into the cognitive impairment arm of the study. The non-AD patients were excluded from the analyses. The rest of the participants were diagnosed to be Alzheimer’s disease based on DSM-IV criteria and their Hachinski ischemic scale [10] were no more than 4.

Most of the patients were diagnosed using psychological tests, information from family, laboratory data, brain structural imaging (X-ray computed tomography or nuclear magnetic resonance imaging) and perfusion single photon emission tomography by dementia specialist, except for participants from Kashiwa cohort.

All procedures were approved by the Ethical Review Board at The University of Tokyo Hospital and The University of Tokyo. The clinical study guidelines of the University of Tokyo, which conform to the Declaration of Helsinki (2013), were strictly adhered to. Healthy volunteers, dementia patients and their families were provided with detailed information about the study, and all provided written informed consent to participate.

### Image preparation

Front-on portrait images were taken of participants wearing a neutral expression. The images were cropped to a square with the face in the middle of the image. Backgrounds were removed so that the AI does not use the background to differentiate cognitive impairment patients from non-dementia patients.

Images of cognitive impairment and healthy participants were divided into 10 groups (group0 ... group9). All images taken of the same participant were included in the same group.

### Selection of models

The network was built using an open-source neural network library, Keras with symbolic tensor manipulation framework, TensorFlow (Google, Mountain View, CA, USA) as a back-end. 5 deep learning models (Xception [11], SENet50 [12], ResNet50 [13], VGG16 [14], simple CNN) with 2 optimizers (SGD, Adam) were tested. Simple CNN was the same network used for the SPECT image study [7]. VGG16, ResNet50, SENet50 were pretrained with VGG-Face [15], and Xception was pretrained with ImageNet [16]. The default settings and parameters for SGD (learning rate = 0.01, momentum=0.9, decay=0.0, nesterov=False) and Adam (learning rate = 0.001, β_1_= 0.9, β_2_= 0.999, =None, decay =0.0, amsgrad=False) were used when being used as optimizers for the simple CNN, but the learning rate was reduced when using the other pretrained models; SGD (learning rate=0.0002, momentum=0.9, decay=0.0, nesterov=False), Adam (learning rate=0.00001, β_1_=0.9, β_2_=0.999, =None, decay=0.0, amsgrad=False). Earlier (about 2/3) layers of Xception, SENet50, ResNet50, and VGG16 except batch normalization layers were frozen. All the layers of simple CNN were trainable. Training image data were augmented (rotation range=15, height shift range=0.03, width shift range=0.03, shear range=5, zoom range=0.1, horizontal flip=True, vertical flip=False, brightness range=[0.3, 1.0], channel shift range=5).

5 models with 2 optimizers were tested with group-base 10-fold cross validation. Learning curves were depicted for 200 epochs. The best model was chosen and the optimum number of epochs determined by considering the accuracy / loss and stability of each model.

### Statistics

The diagnostic and predictive accuracy of the best CNN model was calculated using the group-base 10-fold cross validation. The binary differentiation of cognitive impairment / non-dementia facial images were expressed as “Face AI score”. The scores were obtained by applying an inverse sigmoid function to the output predictive value. Face AI score was evaluated using the ROC curve analysis and AUC on the image-base. Correlations between Face AI score and MMSE / chronological age were assessed. Difference by sex was also examined.

All statistical analyses were performed with python and scipy.stat library.

### Analysis of upper and lower half faces

The faces were divided into upper and lower half faces. Upper half faces and lower half faces were separately trained with the same model as the total faces using the same group-base 10-fold cross validation and the optimum number of epochs determined. The performance was analyzed as described above.
